# Miscellany

**Published:** 1900-02

**Authors:** 


### MISCELLANY.

Proposed Investigation of the Native Drug Plants of the United States.—The secretary of agriculture, Hon. James Wilson, has embraced the following paragraph in his annual report, which, in more particulars than one, is of interest to the medical profession:

The collection of native drug plants in the United States, considered from a purely financial standpoint, aside from medical and humanitarian aspects, involves the expenditure of millions of dollars annually. The commercial extermination of some of the most useful species is already threatened, and doubtless others would be found in the same conditions were the facts known. The price of one native plant, gingseng, our exports of which average more than a million dollars annually, has more than quadrupled in the past thirty years, so that its cultivation, as urged four years ago by this department, has now become profitable. It is clear from this and many similar cases that the native drug industry is capable of either decline or improvement, according to the way in which we handle it.

The Pan-American Medical Congress has recently submitted to me a proposition to coöperate with this department in a technical an statistical investigation and classification of our native drug plants. By accepting this proposal we shall secure, in a research of which we have long felt the need, the cordial assistance and support of an influential association of learned physicians; we shall encourage each of the other American nations, all of which are represented in the Pan-American Medical Congress, to proceed with a similar investigation of their own medical flora; we shall furnish a basis for the remunerative employment of much land and many people, and we shall stimulate the great growth and growing trade in drugs between the countries of North America and South America. I urge the appropriation of $10,000 to enable this department to coöperate in this investigation.

We commend this subject to our readers, and especially to teachers of therapeutics and materia medica. It is also a timely topic to engage the attention of the congress for the revision of the pharmacopeia, which will meet in May, 1900.

---

William Carpenter, of 1260 Fillmore Avenue, Buffalo, was arraigned in Justice Murphy's court, January 12, 1900, on the charge of illegally practising medicine. Dr. John B. Coakley, chairman of the board of censors, made the complaint on behalf of the Medical Society of the County of Erie. He alleged that the defendant advertised that he had for sale certain remedies which were positive cures for specific ailments, and he further claimed that Carpenter had treated several applicants and had accepted payment for his advice. Justice Murphy held Carpenter for the grand jury.

---

Superintendent O'Rourke of Bellevue Hospital, New York, says *The Tribune* received a letter recently from Detroit, in which the writer says he wishes to sell a physician's diploma for $500 to save the owner and his family from being driven from their home. He says the diploma is one of the New York University Medical College of six or seven years ago and that he consents to sell it, a thing he would not countenance under ordinary circumstances. The offense is punishable in this state by a fine of not more than $250 or imprisonment for six months for the first offense, and on conviction of any subsequent offense by a fine of not more than $500, or imprisonment for not less than one year, or both.

---

Governor Candler, of Georgia, is entitled to the thanks of the members of the medical profession throughout the country for vetoing the osteopathy bill passed by the legislature. He disposed of the measure with a few business-like words, and thus saved the medical profession of the empire state of the South from disgrace.

---

Messrs. Scott & Bowne, manufacturing chemists of New York, have placed us under obligation for one of their desk calendars for 1900. This is a renewed courtesy; last year we received one for 1899. The convenience of such an article is very great, and no desk can be regarded as complete without one. We appreciate the courtesy of this famous house. The calendar may be obtained on application to Scott and Bowne, 409-415 Pearl Street, New York.

